# Impact of Socioeconomic Status on the Perception of Accessibility to and Quality of Healthcare Services during the COVID-19 Pandemic among Poles—Pilot Study

**DOI:** 10.3390/ijerph19095734

**Published:** 2022-05-08

**Authors:** Magdalena Tuczyńska, Rafał Staszewski, Maja Matthews-Kozanecka, Ewa Baum

**Affiliations:** 1 SSC of Maxillofacial and Orthopaedics and Orthodontics, Poznan University of Medical Sciences, 61-701 Poznan, Poland; 2 Department of Hypertension, Angiology and Internal Medicine, Poznan University of Medical Sciences, 61-701 Poznan, Poland; rafal.staszewski@ump.edu.pl; 3 Department of Social Sciences and the Humanities, Poznan University of Medical Sciences, 61-701 Poznan, Poland; majamk@ump.edu.pl (M.M.-K.); ebaum@ump.edu.pl (E.B.); 4 Division of Philosophy of Medicine and Bioethics, Poznan University of Medical Sciences, 61-701 Poznan, Poland

**Keywords:** COVID-19, pandemic, socioeconomic status, SES, healthcare

## Abstract

This pilot study was conducted on the reported hypothesis that the COVID-19 pandemic outbreak had an impact on the accessibility and quality of healthcare services and exacerbated socioeconomic inequalities. The aim was to determine whether economic status and education had an impact on the perception of access and quality to healthcare services during the COVID-19 pandemic and whether, according to patients, accessibility and quality had changed significantly compared to the pre-pandemic period in Poland. The study was based on the authors’ questionnaire and the results were statistically analyzed. Two hundred forty-seven feedback responses were received with a responsiveness rate of 93 percent. Statistically significant differences were found when comparing education level and utilization of healthcare services during the COVID-19 pandemic. A comparison of gender and economic situation, and average monthly income found no statistically significant differences. The outbreak of the COVID-19 pandemic has undoubtedly affected the provision of health services in many countries around the world. One result of the pandemic crisis has been widening socioeconomic inequalities among patients.

## 1. Introduction

At the beginning of March 2020, the World Health Organization declared a COVID-19 (Coronavirus Disease) pandemic caused by the SARS-CoV2 virus, first identified in December 2019 in Wuhan, China. The pandemic has affected almost every country globally, leading to the deaths of millions of people worldwide. In Poland, the first confirmed case of COVID-19 was reported by Poland’s Minister of Health on Wednesday 4 March 2020. To limit the spread of the virus, governments have begun to impose restrictions, including the restrictions in movement of people (lockdowns), the need for personal protective equipment (e.g., protective masks), social distancing, limits on shops and state institutions, and much more. Countries have tried to implement various solutions related to pandemics and pathway monitoring on an ongoing basis disease using, for example, drones or special mobile applications, collecting data from areas of interest in real-time. Unfortunately, despite the solutions implemented, it was impossible to avoid an economic crisis [1,2,3,4].

Before the COVID-19 pandemic, accessibility to health services and their utilization was limited for financial, organizational, or social reasons [5]. Scientific reports from various regions of the world (e.g., the United States, Japan, India, Portugal) have indicated an increase in the accessibility and quality of healthcare services among more affluent people. In some cases, people with low socioeconomic status and poor education are not offered the same care as those with higher incomes and quality education, resulting in income-related treatment disparities. Inequalities in access to health care in Poland, mainly to specialist physicians, have also been widely reported in Poland. Research on socioeconomic inequality in the health of Poles has focused on comparing Poland with other countries. Studies have also focused on economic status and gender, education, social determinants of self-assessed health of Polish women and men, and many others [6,7,8,9].

As a result of the pandemic, socioeconomic disparities have widened. In addition, some physicians were diverted to coronavirus testing centers or to hospital wards treating only patients with COVID-19 disease, leaving staff shortages for patients treating chronic conditions or presenting to the emergency room. Patients also had non-urgent appointments rescheduled. These decisions were prompted by recommendations in the Centers for Disease Control and Prevention guidelines. In some cases, medical facilities have tried to divide doctors into those working only onwards with COVID-19 patients and those working to treat patients with other diseases. Furthermore, some patients canceled appointments by themselves for fear of infection. Inequalities also applied to the testing of the population on COVID-19. Apart from the mere lack of specific information on COVID-19 testing guidelines and the small number of testing points, long waiting times for test results were also noted. In addition, there were reports that, concerning socioeconomic status, this time was significantly longer for those with lower socioeconomic status. It was also pointed out that economically disadvantaged patients were twice as likely to present with COVID-19 symptoms to the emergency department than to outpatient facilities. Moreover, socially disadvantaged patients had higher rates of post-discharge healthcare utilization and more often had a re-hospitalization within 30 days after discharge [5,10,11,12,13,14].

Following reports worldwide about inequalities in access to health services related to socioeconomic status, the authors decided to conduct the below pilot study. In addition, based on recent reports on the COVID-19 pandemic, it was hypothesized that the outbreak of the pandemic had an impact on the accessibility and quality of healthcare services and exacerbated socioeconomic inequalities. This pilot study aims to determine whether economic status and education had an impact on the perception of access and quality to healthcare services during the COVID-19 pandemic and whether, according to patients, accessibility and quality had changed significantly compared to the pre-pandemic period in Poland.

## 2. Materials and Methods

### 2.1. Study Design and Participants

This was a pilot study carried out between 8 July and 11 August in 2021, this is the period defined by the Polish government as the third wave of the COVID-19 pandemic. The Bioethics Committee approved it at Poznan University of Medical Sciences (reference number 484/21) in conformity with the Helsinki Declaration guidelines. The study was based on the authors’ questionnaire. Participants in the pilot study were people living in various regions of Poland over the age of 18 who were willing to complete the questionnaire voluntarily. On 8 July 2021, an invitation was distributed via the Google form with a link to the questionnaire and distributed in paper form to patients of the Poznan University of Medical Sciences (Table S1).

### 2.2. Questionnaire

The questionnaire was designed specifically for the study by the authors (Supplementary Material File S1). Questions were prepared in Polish, which is the native language of the respondents. The questionnaire focused on accessibility and quality of health services during the COVID-19 pandemic and was divided into two parts. The first part consisted of general questions, of which there were 12 such as gender, material status, education, etc. The second part focused explicitly on the accessibility and quality of health services. There were ten questions, of which 1 was open-ended and voluntary to answer, 2 required only a “yes” or “no” answer, another 2 required a tick mark on the Visual Analogue Scale (VAS), and the remaining 5, after a “yes” or “no” answer, additionally required a response to sub-questions in the case of an affirmative answer in the main question. The validity and reliability of the questionnaire were established by statistical analysis of scale reliability and Cronbach’s alpha coefficient. It showed that all questions in the questionnaire could remain.

### 2.3. Selected Questions

For this article, individual questions from the questionnaire were selected and statistically analyzed. The first author made the initial selection of questions from the questionnaire, and then the other authors validated or negated the chosen questions. As the article focuses on the impact of socioeconomic status on perceptions of accessibility to and quality of healthcare services, and the questions in the questionnaire also covered a broader range of the subject, the authors chose the questions most relevant to the theme of the article based on subjective assessment. Ultimately, 4 out of 12 questions were selected from the first part, and these were questions about gender, education, economic status, and average monthly income. From the second part, 4 out of 10 questions were selected, and it was 1 question with a “yes” or “no” answer—“Did you use specialist healthcare services during the COVID-19 pandemic?”, 2 questions with answers based on the VAS scale—“How would you rate the accessibility to healthcare services during the COVID-19 pandemic?” and “How would you rate the quality of healthcare services during the COVID-19 pandemic?” and 1 question with a “yes” or “no” answer with a sub-question in case of an affirmative answer—“Did you use healthcare services during the COVID-19 pandemic?” (Main question) and “If yes, please indicate whether these were public, private or primary care healthcare services?” (sub-question). The question on healthcare services referred to services in general, while specialist healthcare services were included as patient visits to doctors specializing in a particular area of medicine, i.e., cardiologist, dentist, gynecologist, etc.

### 2.4. Statistical Analysis

Statistical calculations were performed using Statistica 13 software from TIBCO and PQStat from PQStat Software. The significance level was taken as α = 0.05. The result was considered statistically significant when *p* < α. The Kruskal-Wallis test was calculated to compare variables. The chi2 test of independence or the Fisher-Freeman-Halton test was calculated to examine correlations.

## 3. Results

Two hundred forty-six feedback responses were received from respondents out of 265, which gives a responsiveness rate of 93 percent. The online questionnaire was completed by eighty-two patients and the paper questionnaire was completed by one hundred and sixty-four patients. Four key themes emerged from the analysis. The key themes included: comparison of gender and economic situation, average monthly income, and education (Section 3.1), Impact of economic status, average monthly income and educational background on the accessibility and quality of healthcare services (Section 3.2), the impact of the economic situation on the utilization of healthcare services (Section 3.3), the impact of educational background on the utilization of healthcare services (Section 3.4).

### 3.1. Comparison of Gender and Economic Situation, Average Monthly Income, and Education

1. The question “What is the economic status?” was answered by 240 respondents, of whom 168 were women, and 71 were men. The descriptive analysis showed that 31 respondents are dependent on their parents (21 women—12.5% and 10 men—13.89%), 189 respondents are self-sufficient (135 women—80.36% and 54 men—75.0%) and 20 respondents are partly dependent on their parents/other people (12 women—7.14% and 8 men—11.11%). Pearson’s Chi2 test showed that there was no correlation between gender and the economic situation of the respondents (*p* = 0.546).
2. The question “What is the average monthly income?” was answered by 238 respondents (165 women and 73 men). Eighty-six respondents earn above the national average (54 women—32.73% and 32 men—43.84%), 57 people earn at the same level as the national average (44 women—26.67% and 13 men—17.81%), 73 people earn below the national average (52 women—31.52% and 21 men—28.77%), 22 respondents declared they have no income at all (15 women—9.09% and 7 men—9.59%). Pearson’s Chi2 test showed that there was no correlation between gender and the average monthly income of the respondents (*p* = 0.318).
3. The question “What is your educational background?” was answered by 230 respondents (159 women and 71 men). More than a half (i.e., 146 respondents) had higher education (110 women—69.18% and 36 men—50.70%), 59 people had secondary education (37 women—23.27% and 22 men—30.99%), 23 respondents had lower secondary education (11 women—6.92% and 12 men—16.90%), only two respondents had primary education (1 woman—0.63% and 1 man—1.41%). The Fisher-Freeman-Halton test showed a correlation (*p* = 0.017), which means that female respondents had a higher education than male respondents (Table 1).

### 3.2. Impact of Economic Status, Average Monthly Income, and Educational Background on the Accessibility and Quality of Healthcare Services

In all cases analyzed below, the Kruksal-Wallis test was performed to investigate whether economic status, average monthly income, or educational background affected the accessibility and quality of healthcare services during the COVID-19 pandemic in Poland (Table 2).

1. In none of the analyzed cases concerning economic status, i.e., in the group of self-sufficient respondents, parent’s/other’s dependent and partially dependent parent’s/other people showed no significant statistical differences for both accessibility and quality assessment of health services before and during the COVID-19 pandemic.
2. Also, when analyzing the impact of average monthly income, in none of the analyses cases, i.e., in the group of people earning below the national average, equal to the national average, above the national average, and having no income at all, no significant statistical differences for both accessibility and quality assessment to healthcare services before and during the COVID-19 pandemic were observed.
3. Comparable results were also obtained to analyze the impact of education on accessibility and quality of healthcare services during the COVID-19 pandemic. There were no statistically significant differences in none of the groups, i.e., with higher education, secondary education, lower secondary education, and primary education.

### 3.3. The Impact of Economic Status on the Utilisation of Healthcare Services

The analysis of the impact of economic status on the utilization of general and specialized healthcare services was performed using the Fisher-Freeman-Halton test. The research was based on 241 responses. No statistically significant differences were found for the utilization of general (*p* = 0.528) and specialized (*p* = 0.412) healthcare services. Sub-questionnaire analysis showed that only for primary care services did economic status affect the utilization of these services (*p* = 0.041). This means those self-supporting individuals were more likely to use primary care healthcare services. No statistically significant differences were found in the remaining cases, i.e., the utilization of public (*p* = 0.825) or private (*p* = 0.178) sector healthcare services (Table 3).

### 3.4. The Impact of Educational Background on the Utilisation of Healthcare Services

The analysis of the impact of educational background on the utilization of general and specialized healthcare services was performed using the Fisher-Freeman-Halton test. The analysis was based on 230 responses. Statistically significant differences were obtained in all cases analyzed. Respondents with higher education were more likely to utilize general healthcare services (*p* = 0.000), during the COVID-19 pandemic, including both public (*p* = 0.007), private (*p* = 0.000), and primary healthcare services (*p* = 0.020). Respondents with higher education were also more likely to utilize specialist healthcare services (*p* = 0.020) (Table 3).

## 4. Discussion

Based on reports from the world, it can be concluded that socioeconomic status impacts health care. People with lower status are thought to have limited access to health care due to the cost and insurance coverage, wait longer to be admitted for a medical appointment, and receive fewer diagnostic tests and medications for many chronic diseases, in addition, poorer neighborhoods have more users and less service time per user than in wealthier areas. Moreover, patients’ belief that their care is somehow inferior often results in a loss of trust in the health care system or the healthcare providers [15,16,17]. During the COVID-19 pandemic, access to adequate healthcare, medicines, and vaccines was limited, as was the number of hospital admissions, surgeries, and non-invasive medical procedures performed [18,19]. Collaboration between doctors and pharmacists has proven to be one solution to that problem. The implementation of such collaboration provided better control over potential disease-drug interactions and had a positive impact on patient care and clinical outcomes [20]. Our questionnaire compared the impact of socioeconomic status on the utilization, accessibility, and quality of healthcare services and did not reveal statistically significant differences in all cases. These results do not differ significantly from other reports from around the world. However, it should be noted that similarities or differences between our questionnaire and other results worldwide may be due to similar or completely different economic situations in a given country and Poland.

A study of US patients conducted during the COVID-19 pandemic found no statistically significant income level impact on access to healthcare services [21]. Moreover, statistical analysis of our data showed no differences in access to health services during the COVID-19 pandemic by average income earned. It can be explained that people in low socioeconomic groups tend to be younger and healthier. Such people are less likely to seek healthcare or not to report a delay in accessing it [21]. On the other hand, a study of people aged over 40 in Malaysia, where more than 80% of respondents reported meager income, revealed that almost twice as many respondents used healthcare services during the peak of the COVID-19 pandemic as during the lifting of restrictions. Additionally, it was found that despite the increase associated with the lifting of the restrictions, the percentage of utilization of healthcare services was still at a low level [22]. Higher socioeconomic status becomes more relevant as the population ages. It becomes apparent that the health of older people with a low socioeconomic status is poorer in comparison to wealthier people [7]. Moreover, people with low socioeconomic status are considered to wait longer for a medical appointment, are more likely to have to forego treatment for financial reasons and have limited access to medicines [23,24,25].

Interesting results were obtained when comparing the impact of education on the utilization of healthcare services. While the more frequent utilization of private healthcare services by respondents with higher education is not surprising, the more frequent utilization of public and primary healthcare services by these people is curious. Primary Health Care in Poland is a part of the health care system that provides access to comprehensive healthcare services at the place of residence. Patients can access free medical services, including medical and pediatric consultations, nursing and midwife services, and telephone consultations. Primary Health Care is a medical point based on a public health insurance system that National Health Fund represents. The perception of the quality of primary health care in Poland before the pandemic was not elevated. However, in terms of accessibility, it was considered a major aspect of primary health care services, next to comprehensiveness and continuity. The more frequent utilization of primary health care services by people with higher education is surprising. The quality of these services had already been rated low in earlier studies before the pandemic. Higher education is associated with higher earnings so that people can afford private care and yet often choose public care. One explanation for this may be that private facilities completely suspended their activities during the pandemic, while primary health care facilities reduced but did not completely shut down their functioning. A descriptive survey of four different medical facilities in Poland showed that the reduction in primary care admissions was small (around 10%) compared with the pre-pandemic period. Still, it should be noted that most of these facilities increased the number of visits provided by telemedicine [26,27,28]. Similar results were obtained in a population-based cross-sectional study conducted in Hungary. It showed that during the pandemic, people with higher education were more likely to use specialist healthcare services, which is in agreement with our results, while they were less likely to use GP services [29]. Interesting results were presented in a survey of households in Togo. Based on the respondents’ answers, it was found that people with higher education were less likely to use healthcare services in medical facilities due to fear of infection. Self-treatment at home was used as an alternative to visiting a medical facility. The low proportion of utilization of healthcare services was assigned by the author of the study to the conversion of medical facilities to hospitalized COVID-19 [30].

During the COVID-19 pandemic, telemedicine developed rapidly. Patients received appointments without having to leave their homes via a telephone or video call. The respondents’ good accessibility and quality of health services maybe because they could access appointments online. Therefore, after reviewing the results of the pilot study, the authors of the study planned to modify the questions in the questionnaire to distinguish teleconsultations from in-person visits [31,32]. Such a distinction would enrich further research, particularly in relation to access to telemedicine and socioeconomic status, especially as there have been reports in the literature that people of lower socioeconomic status have poorer access to this form of healthcare services [33,34]. In overall, it is worth highlighting that telemedicine is a safe and useful tool of communication with a patient, which increases access to medical care. In Poland, during the COVID-19 pandemic, most medical facilities introduced teleconsultation, allowing patients to consult a doctor by phone or computer. Although this form of visit gained importance during the pandemic because it was easily accessible for most patients, its quality was not always satisfactory. In addition to teleconsultation, telemonitoring of patients after myocardial infarction has developed, including e-oximeter, allowing for monitoring of heart rhythm and blood saturation [35,36,37].

The lack of direct access to physicians during the COVID-19 pandemic, also contributed to the popularization of self-medication. Self-medication is considered part of a broader self-care process that motivates individuals to take action to improve their health, as well as prevent and treat disease. One of the advantages of self-treatment is that it reduces the economic burden on patients and the health care system. However, the consequences of inappropriate and unnecessary use of self-medication cannot be underestimated, as it can lead to adverse effects, delay in reporting to a specialist, masking of symptoms, incorrect diagnosis, drug interactions, and increased expenditure on medicines. Limited access to a doctor also influenced the behavior of Poles related to self-treatment. A survey conducted among Poles focusing on the 3-month lockdown period related to the COVID-19 pandemic showed that the self-treatment behavior of some respondents changed. Respondents declared buying prescription drugs without a clear need, not going to a doctor’s appointment, as well as people who usually did not take drugs preventively, did so, as well as taking prescription drugs without consulting a doctor if they had not done so before [38,39,40,41].

The pilot survey we conducted has its advantages; namely, it was based on an original survey questionnaire, the respondents were patients from different regions of Poland, the questionnaire was anonymous, and it had a high response rate. On the other hand, the disadvantages include the lack of representativeness, inability to reach selected groups of patients, e.g., in nursing homes or palliative care, exclusion of pediatric patients, and the possibility of mode effect (as the analysis of the results concerned both questionnaires completed on paper and online), data collected during only one pandemic wave and lack of a specific distinction between face-to-face visits and in the form of teleconsultations.

Despite its limitations, the study provides a compelling example of the impact of socioeconomic status on the accessibility and quality of health services during the difficult period of the COVID-19 pandemic. The research team will use the findings to implement improvements to the questionnaire, as well as data collection. It is planned to expand the group of respondents, perform an extended data analysis based also on other aspects of the questionnaire not included in the pilot study, and obtain responses from respondents in other European countries for comparison.

## 5. Conclusions

The outbreak of the COVID-19 pandemic has undoubtedly affected the provision of health services in many countries around the world. One result of the pandemic crisis has been widening socioeconomic inequalities among patients. While many reports from around the world have suggested that access to and quality of services has deteriorated during the COVID-19 pandemic, the results of our pilot study did not show that socioeconomic status had a significant impact on access, quality, and utilization of healthcare services. Only in the case of the effect of education, it was shown that people with higher education more often used all types of health services. Yet, the research requires continuation among a more significant number of patients, as well as obtaining feedback from questionnaires from other regions of the world to compare the results.

## Figures and Tables

**Table 1:** Percentage distribution of respondents.

Variable		Gender		*p* Value
		Women	Men	
Economic status	dependent on parents	21 (12.50%)	10 (13.89%)	0.546
	self-sufficient	135 (80.36%)	54 (75.00%)	
	partly dependent on parents/other people	12 (7.14%)	8 (11.11%)	
Average monthly income	above the national average	54 (32.73%)	32 (43.84%)	0.318
	same level as the national average	44 (26.67%)	13 (17.81%)	
	below the national average	52 (31.52%)	21 (28.77%)	
	no income	15 (9.09%)	7 (9.59%)	
Educational background	higher education	111 (69.18%)	36 (50.70%)	0.017 *
	secondary education	37(23.27%)	22 (30.99%)	
	lower secondary education	11 (6.92%)	12 (16.90%)	
	primary education	1 (0.63%)	1 (1.41%)	

* statistically significant result.

**Table 2:** Statistical significance for assessing the accessibility and quality of healthcare services.

	Economic Status	Average Monthly Income	Educational Background
Accessibility before pandemic	*p* = 0.757	*p* = 0.312	*p* = 0.271
Accessibility during pandemic	*p* = 0.329	*p* = 0.219	*p* = 0.935
Change in accessibility rating	*p* = 0.078 ^1^	*p* = 0.225	*p* = 0.112
Quality before pandemic	*p* = 0.699	*p* = 0.686	*p* = 0.674
Quality during pandemic	*p* = 0.446	*p* = 0.192	*p* = 0.510
Change in quality rating	*p* = 0.357	*p* = 0.084 ^1^	*p* = 0.443

^1^ result on the statistical significance borderline.

**Table 3:** Statistical significance for assessing the utilization of healthcare services during the COVID-19 pandemic (Table 2).

	Economic Status	Educational Background
General healthcare	*p* = 0.528	*p* = 0.000 ^1^
Public healthcare	*p* = 0.825	*p* = 0.007 ^1^
Private sector healthcare	*p* = 0.178	*p* = 0.000 ^1^
Primary care healthcare	*p* = 0.041 ^1^	*p* = 0.020 ^1^
Specialist healthcare	*p* = 0.412	*p*=0.020 ^1^

^1^ statistically significant result.

## Data Availability

Data are available on request from the authors.

## Supplementary Materials

The following supporting information can be downloaded at: https://www.mdpi.com/article/10.3390/ijerph19095734/s1, Table S1: Main sociodemographic characteristics; File S1: Survey questionnaire on the access to Healthcare Services during the COVID-19 pandemic.
