# Supplementary material for: Impact of Socioeconomic Status on the Perception of Accessibility to and Quality of Healthcare Services during the COVID-19 Pandemic among Poles—Pilot Study
Source: Int J Environ Res Public Health. 2022 May 8;19(9):5734. doi: 10.3390/ijerph19095734 (PMC9104779; doi:10.3390/ijerph19095734)
Supplement: Supplementary file 1 [file ijerph-19-05734-s001.zip › File S1.pdf]

SURVEY QUESTIONNAIRE ON THE ACCESS TO HEALTHCARE SERVICES DURING THE COVID-19 PANDEMIC

Dear Participant,

I kindly ask you to take part in the study and fill in the questionnaire. The purpose of this survey is to learn the opinions of adult patients on access to healthcare services during the COVID-19 pandemic. The survey is anonymous. After completing the questionnaire, a signature is not required. The conclusions drawn as a result of the collective survey will be used for scientific studies. Before filling out the questionnaire, please read it carefully. When answering the following questions, select the answer(s) in line with your personal views from among the answers provided, or enter the answer in the right place. Please answer each question.

By completing the questionnaire, you agree to participate in the study.

Thank you for completing the survey carefully and sincerely.

Magdalena Tuczyńska

METRIC:
Please mark the field of the chosen answer - X

FEMALE - ❑ MALE - ❑

**What is your place of residence:**<br><br>• **city with up to 50.000 residents** ❑<br>• **city with over 50.000 up to 100.000 residents** ❑<br>• **city with over 100.000 residents** ❑<br>• **village** ❑<br><br>**Do you live:**<br><br>• **alone** ❑<br>• **with wife/husband** ❑<br>• **with a partner** ❑<br>• **with family** ❑<br>• **other** ❑<br><br>**What is your current country of residence?**<br><br>…………………………………………………………	**How old are you:**<br><br>**men:**<br>• **18-64 years old** ❑<br>• **over 65** ❑<br>**women:**<br>• **18-59 years old** ❑<br>• **over 60** ❑<br><br>**Are you a person with a specific disability:**<br><br>• **yes** ❑<br>• **no** ❑

What is your situation:	What is your education:
• I am financially self-sufficient ❑<br>• I am dependent on my parents or other people ❑<br>• I am partially dependent on my parents or other people ❑<br><br>**How much is your monthly income:**<br><br>• below the national average ❑<br>• equal to the national average ❑<br>• above the national average ❑<br>• I don't have any income ❑	• primary/ elementary ❑<br>• lower secondary ❑<br>• upper secondary ❑<br>• post-secondary ❑<br>• higher:<br>Bachelor's Degree ❑<br>Master's Degree ❑<br>PhD/ Advanced Professional Degree and above ❑

Please specify your health self-assessment:	What is your religious affiliation:
• very good ❑<br>• good ❑<br>• neither good nor bad ❑<br>• bad ❑<br>• very bad ❑	……………………………………………………<br><br>**What is the importance of religion in your life:**<br><br>• is crucial ❑<br>• plays a big role ❑<br>• hard to say ❑<br>• slight ❑<br>• not important ❑

1. Did you use medical services during the COVID-19 pandemic?

❑ yes
❑ no

If so, please mark which (you can choose more than one answer):

❑ state-funded medical services
❑ fully paid medical services
❑ primary healthcare services
❑ other, please specify which
……………………………………………………………………………………………

2. Have you had COVID-19? :

❑ yes
❑ no

If so, was there a problem with (you can mark more than one answer):

❑ making an appointment with a primary care physician
❑ providing direct medical advice
❑ receiving medical advice by phone
❑ other, please specify which
………………………………………………………………………………………………

3. Did you use any specialist services during the COVID-19 pandemic? :

❑ yes
❑ no

If so, please mark which:

❑ cardiology
❑ general practice
❑ ENT / laryngology
❑ psychiatric
❑ psychological
❑ dermatological
❑ pulmonary
❑ neurological
❑ orthopedic
❑ obstetrics and gynecological
❑ urological
❑ endocrine
❑ oncology
❑ ophthalmic
❑ dental
❑ nursing
❑ physiotherapeutic
❑ aesthetic medicine
❑ other, please specify which:
………………………………………………………………………………………………

4. Did you have any problems with receiving a referral for diagnostic tests during the COVID-19 pandemic?:

❑ yes
❑ no

If so, please mark with which (you can mark more than one answer):

- ❑ X-ray
- ❑ magnetic resonance imaging (MRI scan)
- ❑ computed tomography (CT scan)
- ❑ ultrasound examination (USG)
- ❑ mammography
- ❑ laboratory tests
- ❑ other, please specify which:

………………………………………………………………………………………

5. Was any of your relatives or friends in the COVID-19 ward during the COVID-19 pandemic?:

- ❑ yes
- ❑ no

If so, please mark if there was a possibility to (you can mark more than one answer):

- ❑ visit them
- ❑ call them
- ❑ make a video call with them
- ❑ hand over the package to them
- ❑ get an information about their medical condition
- ❑ other, please specify what

………………………………………………………………………………………

6. Did you cancel a medical appointment during the COVID-19 pandemic?:

- ❑ yes
- ❑ no

If so, mark the reason of it (you can mark more than one answer):

- ❑ fear of being infected
- ❑ quarantine
- ❑ fear of using public transport
- ❑ other, please specify which:

………………………………………………………………………………………

If so, please mark which other forms of treatment you have used (you can mark more than one answer):

- ❑ pharmacist's advice at a pharmacy
- ❑ home remedies
- ❑ advice via the Internet (internet search, internet forum)
- ❑ other, please specify which:

………………………………………………………………………………………

7. Do you think that Patients' Rights were respected during the COVID-19 pandemic?:

❑ yes
❑ no

8. Has your spiritual life (mindfulness, religious practices, meditation, focus, developing your own passions, supporting loved ones and / or using other internal resources) helped you during the COVID-19 pandemic?:

❑ yes
❑ no

9. How do you assess the access of health services during the COVID-19 pandemic?:

BEFORE THE PANDEMIC

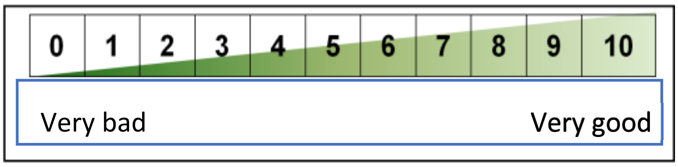

DURING THE PANDEMIC

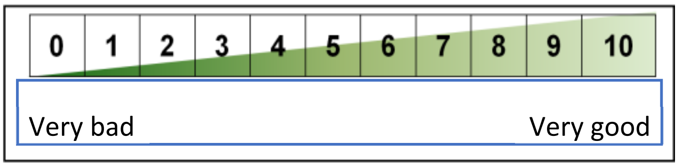

10. How would you rate the quality of healthcare services during the COVID-19 pandemic?:

BEFORE THE PANDEMIC

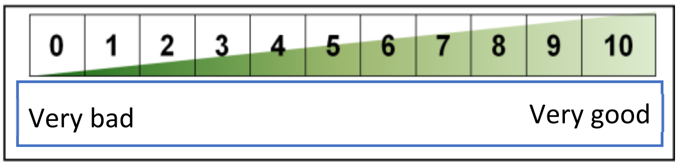

DURING THE PANDEMIC

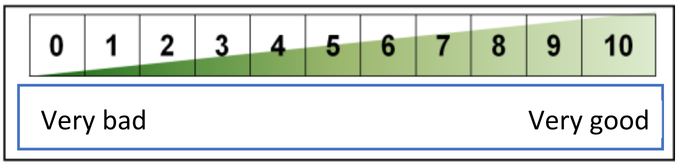

Open question:

If you believe that there have been significant changes in the quality of services provided, please indicate them below:

……

……
