# Supplementary material for: Impact of Socioeconomic Status on the Perception of Accessibility to and Quality of Healthcare Services during the COVID-19 Pandemic among Poles—Pilot Study
Source: Int J Environ Res Public Health. 2022 May 8;19(9):5734. doi: 10.3390/ijerph19095734 (PMC9104779; doi:10.3390/ijerph19095734)
Supplement: Supplementary file 1 [file ijerph-19-05734-s001.zip › Table S1.pdf]

Supplementary table
Table S1. Main sociodemographic characteristics.

**Variable**		**n**	**%**
**Sex**	Women	168	69
	Men	77	31
**Economic status**	Dependent on parents	20	8
	Self sufficient	190	79
	Partly dependent on parents/others	31	134
**Average monthly income**	Above average	86	36
	Same as average	57	24
	Below average	74	31
	No income	22	9
**Educational background**	Higher	147	64
	Secondary	59	25
	Lower	23	10
	Primary	2	1
